# Identification the ferroptosis-related gene signature in patients with esophageal adenocarcinoma

**DOI:** 10.1186/s12935-021-01821-2

**Published:** 2021-02-18

**Authors:** Lei Zhu, Fugui Yang, Lingwei Wang, Lin Dong, Zhiyuan Huang, Guangxue Wang, Guohan Chen, Qinchuan Li

**Affiliations:** 1 Department of Thoracic Surgery, Shanghai East Hospital, Tongji University School of Medicine, Shanghai, 200120 China; 2 Research Center for Translational Medicine, Shanghai East Hospital, Tongji University School of Medicine, Shanghai, 200120 China; 3 Department of Neurosurgery, Shanghai East Hospital, Tongji University School of Medicine, Shanghai, 200120 China

**Keywords:** Esophageal adenocarcinoma, Ferroptosis, Bioinformatics analysis, TCGA

## Abstract

**Background:**

Ferroptosis is a recently recognized non-apoptotic cell death that is distinct from the apoptosis, necroptosis and pyroptosis. Considerable studies have demonstrated ferroptosis is involved in the biological process of various cancers. However, the role of ferroptosis in esophageal adenocarcinoma (EAC) remains unclear. This study aims to explore the ferroptosis-related genes (FRG) expression profiles and their prognostic values in EAC.

**Methods:**

The FRG data and clinical information were downloaded from The Cancer Genome Atlas (TCGA) database. Univariate and multivariate cox regressions were used to identify the prognostic FRG, and the predictive ROC model was established using the independent risk factors. GO and KEGG enrichment analyses were performed to investigate the bioinformatics functions of significantly different genes (SDG) of ferroptosis. Additionally, the correlations of ferroptosis and immune cells were assessed through the single-sample gene set enrichment analysis (ssGSEA) and TIMER database. Finally, SDG were verified in clinical EAC specimens and normal esophageal mucosal tissues.

**Results:**

Twenty-eight significantly different FRG were screened from 78 EAC and 9 normal tissues. Enrichment analyses showed these SDG were mainly related to the iron-related pathways and metabolisms of ferroptosis. Gene network demonstrated the TP53, G6PD, NFE2L2 and PTGS2 were the hub genes in the biology of ferroptosis. Cox regression analyses demonstrated four FRG (CARS1, GCLM, GLS2 and EMC2) had prognostic values for overall survival (OS) (all P < 0.05). ROC curve showed better predictive ability using the risk score (AUC = 0.744). Immune cell enrichment analysis demonstrated that the types of immune cells and their expression levels in the high-risk group were significant different with those in the low-risk group (all P < 0.05). The experimental results confirmed the ALOX5, NOX1 were upregulated and the MT1G was downregulated in the EAC tissues compared with the normal esophageal mucosal tissues (all P < 0.05).

**Conclusions:**

We identified differently expressed ferroptosis-related genes that may involve in EAC. These genes have significant values in predicting the patients’ OS and targeting ferroptosis may be an alternative for therapy. Further studies are necessary to verify these results of our study.

## Background

Esophageal cancer is the eighth most common malignancy in the world, which accounts for approximately 570,000 new cases and 500,000 deaths per year worldwide [1]. Esophageal adenocarcinoma (EAC) is one of the main pathological types, characterized by high incidence and poor prognosis [2]. It’s estimated that the proportion of patients with EAC nearly doubled, from 35 to 61% over the past 30 years in the western countries, with a global incidence rate of 0.7 per 100,000 person-years [3, 4]. Despite the tremendous progress has been made in therapy, including esophagectomy, radiation, chemotherapy and molecular targeted drugs, the 5-year survival rate still remains less 20% [3]. Therefore, an optimal management aiding in the early detection and therapeutic improvements is imperative. The latest studies have shown ferroptosis, a non-apoptotic programmed cell death, has emerged to play an important role in tumor biology and may open up new opportunities for EAC [5, 6].

Ferroptosis, firstly proposed by Stockwell BR lab in 2012, is a necrotic cell death modality that is morphologically, biochemically and genetically distinct from apoptosis, necrosis and autophagy [7]. This process is marked by the accumulation of reactive oxygen species (ROS) via an iron-dependent mechanism [7]. An initial characterization of the mechanism triggering ferroptosis is the cysteine depletion, which leads to the exhaustion of glutathione (GSH) in the intracellular [7]. Hence fore, it’s conceivable a complex interplay that regulates the different cancer cells susceptibilities to ferroptosis should be a fruitful area in cancer research. A large amount studies have confirmed many genes are involved in the initiation and execution of ferroptosis in cancers [8–10]. These discoveries shed light on the tumor ferroptotic plasticity and provide insights into how ferroptosis is associated with the persistence, dedifferentiation and expansion of cancer cells. In addition, evidences from several researches demonstrate the ferroptotic cells could interact with NK cells, CD8+ T cells and other immune cells by releasing some chemotaxis, thus modulating the anticancer immunity [11–13]. However, it’s undeniable that much less clear about how the ferroptosis is elaborately regulated and it is far from applying the ferroptosis to cancer therapy.

A better understanding of ferroptosis is critical for immune surveillance and therapeutic management, as well as paving ways for further explorations. To our best known, there is scarce study exploring the link between the ferroptosis and EAC, and their relationships with survival in EAC patients have never been studied. In this study, we aim to investigate the ferroptosis-related genes (FRG) expression profiles and their values in the prognosis in EAC through the bioinformatics analysis. The results were verified in clinical specimens through polymerase chain reaction (PCR) and immunohistochemistry (IHC).

## Methods

### Acquisition of gene expression and clinical data

Gene expressions of transcriptome profiles and clinical data of EAC patients were downloaded from The Cancer Genome Atlas (TCGA) website at https://portal.gdc.cancer.gov/. The raw data of gene expression was normalized using the “limma” R package in R software. The 60 FRG were retrieved from previous published literatures and available in the Additional file 1: Table S1 [14–17].

### Identification of significantly different and prognostic genes

The significantly different genes (SDG) were identified using the “limma” R package with the Wilcoxon test. The cut-off values were determined according to the parameters, P < 0.05 and false discovery rate (FDR) < 0.05.

Univariate and multivariate cox regressions were used to evaluate the relationships between the SDG and the patients’ overall survival (OS). Patients were divided into high-risk and low-risk groups according to the risk score. The risk score was calculated by the following formula: ${\text{risk}}\;{\text{score}} = \sum\nolimits_{\left( {n = 1} \right)}^j {Coef\;j*{Xj},}$, with Coef j representing the coefficient and Xj representing the relative expression levels of each SDG standardized by z-score.

### Interaction network and enrichment analysis

An interaction network of SDG was performed at the STRING website (http://string-db.org/cgi/input.pl). Then, we also explored their correlations using the R software.

Next, the functional enrichment analysis of Gene Ontology (GO), including the biological process, cellular component, and molecular function was performed by R software. The Kyoto Encyclopedia of Genes and Genomes (KEGG) analysis was also done using the same tool.

### Development of ROC curves

Then, we combined the prognostic FRG with clinical information using the univariate cox regression. Significant prognostic factors (P < 0.05) were enrolled into multivariate cox regression to identify the independent prognostic risk factors. The receiver operating characteristic (ROC) analysis was used to examine the sensitivity and specificity of survival prediction using the independent risk factors. The area under curve (AUC) of the ROC ranges from 0.5 to 1, with near 1 indicating perfect predictive ability and 0.5 indicating without predictive ability.

### Immune cells and ferroptosis

We further evaluated the infiltrating scores of 16 immune cells and the activities of 13 immune-related pathways with the single-sample gene set enrichment analysis (ssGSEA) in the "gsva" R package [18]. The annotated gene set file is provided in Additional file 2: Table S2. Moreover, the relationships between immune cells and prognostic genes were explored through TIMER database (https://cistrome.shinyapps.io/timer/).

### Experimental validation

To verify SDG expression profiles in EAC and normal tissues, we conducted the experimental validation in 15 EAC patients’ specimens who received esophagectomy from 2019 January to 2020 June in Shanghai East Hospital, Tongji University School of Medicine. Ten normal esophageal mucosal tissues were used as control. This study was approved by the Internal Review Board of Shanghai East Hospital, Tongji University School of Medicine.

Total RNA from EAC specimens and normal tissues was purified using RNAiso plus (Takara, Dalian, China). Complementary DNA (cDNA) was synthesized from 1 μg of total RNA using a PrimeScript® RT reagent Kit with gDNA (genomic DNA) Eraser (Takara). TB Green® Premix Ex Taq® II kit (Takara) was used to detect the indicated RNA levels on the QuantStudio Real-Time PCR System (Applied Biosystems, USA) or the CFX96 Real-Time System (Bio-Rad, USA). The relative expression levels of the candidate FRG were normalized to endogenous GAPDH (glyceraldehyde-3-phosphate dehydrogenase). The primers synthesized and by GENEWIZ company, Suzhou, China. The primers are listed in Additional file 3: Table S3.

Next, paraffin-embedded sections were stained for IHC analysis at 4 µm thickness. IHC was performed with antibodies for NOX1 (1:150, ab131088, abcam), PTGS2 (1:50, ab169782, abcam), ALOX5 (1:100, ab169755, abcam) and TFRC (1:500, ab214039, abcam). Slides were incubated with primary antibody for 40 min at 37 °C, and secondary antibody for 20 min at 37 °C. Then, slides were incubated in streptavidin–horseradish peroxidase (SA-HRP) D for 16 min at 37 °C. The substrate, 3,3′-diaminobenzidine tetrahydrochloride (DAB) H_2_O_2_ was added for 8 min followed by hematoxylin and bluing reagent counterstaining at 37 °C. Vectastain Elite Kit (Vector Laboratories, Burlingame, CA, USA) was used for visualization of immunostaining. The absence of protein expression was defined when none of cells showed staining. The protein expression was designated as the presence of staining of tumor cells, irrespective of the proportion or intensity.

### Statistical analysis

Student’s t-test was used to compare gene expression differences between tumor and normal tissues. Univariate and multivariate cox regression analyses were used to evaluate the correlations between the factors and patients’ OS. Log-rank test was used to compare the survival differences between high and low-risk groups. Kaplan–Meier curve was implemented to visualize the survival. Mann–Whitney test with P values adjusted by the BH method was used to compare the ssGSEA scores of immune cells or pathways between the high-risk and low-risk groups. All the statistics were done using the R software (version 4.0.2). P value < 0.05 was set as statistically significant for all the analyses.

## Results

### Identification of SDG and patients’ clinical data

**Fig. 1:**
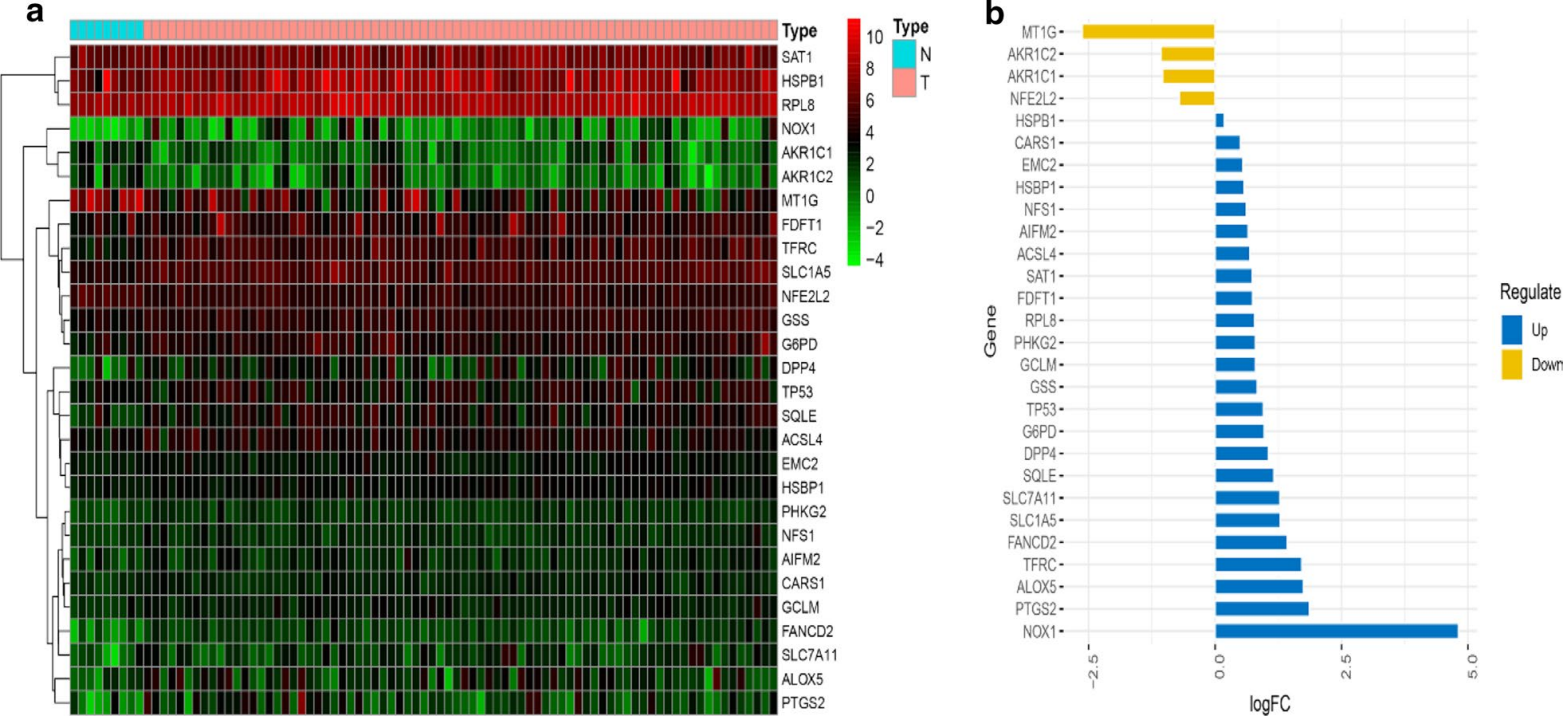
Identification of SDG in normal and EAC tissues. **a** heatmap of SDG. Green represents down-regulation and red represents up-regulation of genes. **b** deviation plot of SDG. Yellow bars represent 4 down-regulated genes; blue bars represent 24 up-regulated genes. *N* normal, *T* tumor (EAC)

A total of 9 normal and 78 EAC samples with gene expression profiles and clinical information were retrieved from TCGA dataset (Additional files 4 and 5). After analysis, there were 28 significantly different ferroptosis-related genes between normal and EAC samples. Among these, four FRG (AKR1C1, AKR1C2, MT1G and NFE2L2) were down-regulated in EAC tissues compared with normal tissue, other 24 genes were up-regulated (Table 1). The heatmap and deviation plots are shown in Fig. 1a, b.

**Table 1:** significantly different FRG expression levels in EAC and normal tissue

Gene	Normal	EAC	logFC	P value	FDR
ACSL4	11.274	18.158	0.688	0.020	0.045
AIFM2	3.554	5.584	0.652	0.022	0.045
AKR1C1	6.188	3.015	− 1.037	0.006	0.019
AKR1C2	6.788	3.245	− 1.065	0.019	0.045
ALOX5	2.671	8.927	1.741	0.021	0.045
CARS1	4.068	5.739	0.496	0.006	0.019
DPP4	6.142	12.714	1.050	0.005	0.019
EMC2	5.861	8.602	0.553	0.001	0.006
FANCD2	1.290	3.465	1.426	0.001	0.004
FDFT1	28.431	47.563	0.742	0.025	0.050
G6PD	14.208	27.870	0.972	0.006	0.019
GCLM	4.080	7.077	0.795	0.001	0.004
GSS	16.051	28.628	0.835	0.000	0.003
HSBP1	6.611	9.820	0.571	0.001	0.004
HSPB1	324.993	369.728	0.186	0.011	0.029
MT1G	336.078	54.328	− 2.629	0.000	0.003
NFE2L2	45.899	28.153	− 0.705	0.000	0.003
NFS1	3.264	5.003	0.616	0.010	0.028
NOX1	0.200	5.601	4.809	0.000	0.002
PHKG2	2.393	4.149	0.794	0.000	0.003
PTGS2	2.205	8.034	1.865	0.002	0.009
RPL8	259.463	445.943	0.781	0.001	0.006
SAT1	94.001	155.854	0.729	0.016	0.039
SLC1A5	22.655	55.248	1.286	0.000	0.001
SLC7A11	2.194	5.322	1.279	0.010	0.028
SQLE	8.165	18.257	1.161	0.001	0.006
TFRC	12.433	40.774	1.713	0.000	0.002
TP53	9.948	19.273	0.954	0.020	0.045

*LogFC* log fold change, *FDR* false discovery rate

### Functional enrichment analysis of SDG

**Fig. 2:**
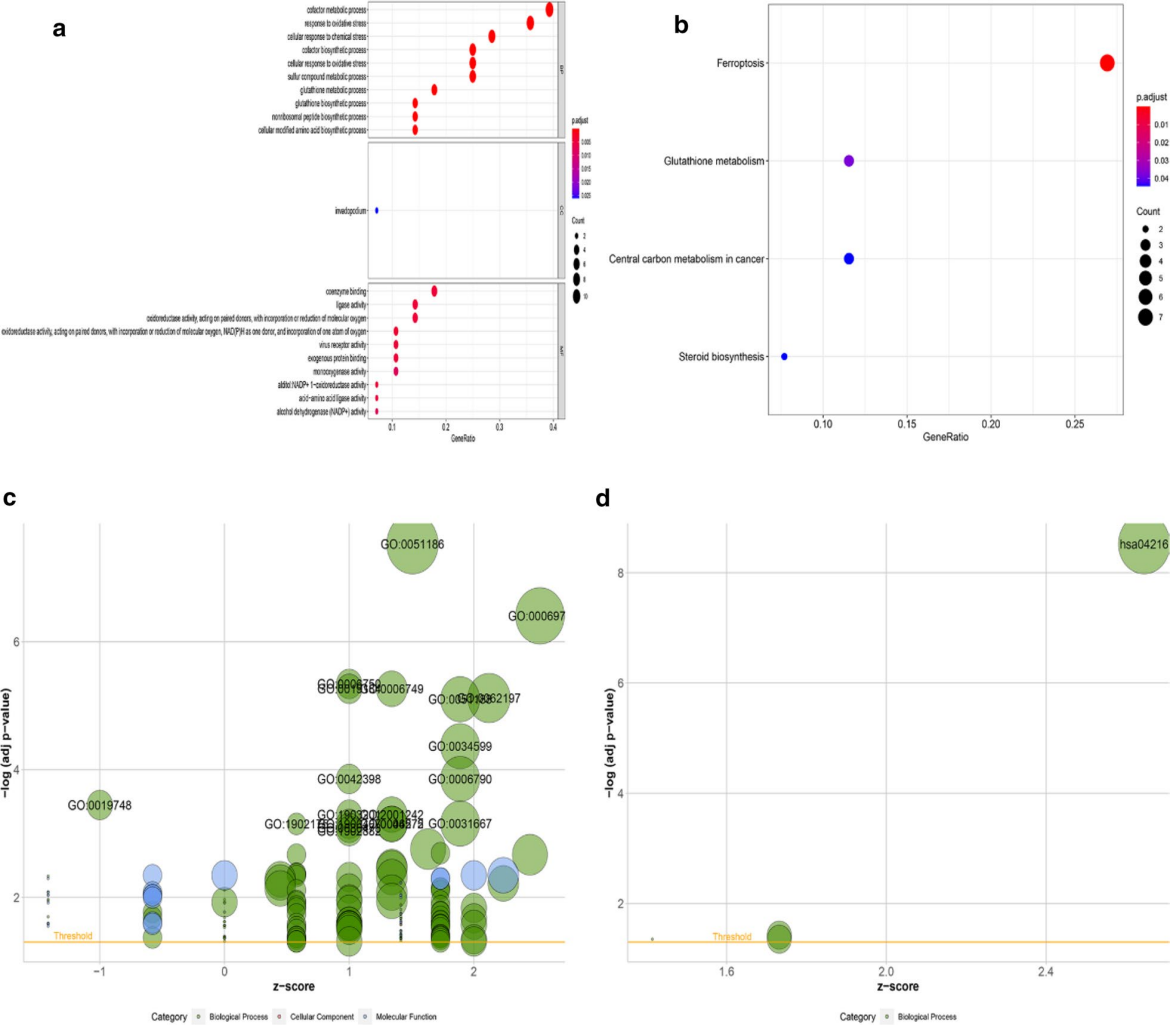
Representative results of GO (**a**, **c**) and KEGG (**b**, **d**). Bubble plots of GO (**a**) and KEGG (**b**) analyses. Results of GO (**c**) and KEGG (**d**) in the form of gene ID. The larger bubble indicates the more obvious enrichment

To elucidate the biological functions and pathways of SDG in ferroptosis, the 28 genes were used to functional enrichment analysis. The GO results showed that SDG were enriched in iron-related pathways, such as metabolic and oxidative process. KEGG analysis showed the SDG were closely enriched in ferroptosis, including the GSH metabolism, oxidative reaction and biosynthesis (Fig. 2a–d).

### Interactions and correlations of SDG

**Fig. 3:**
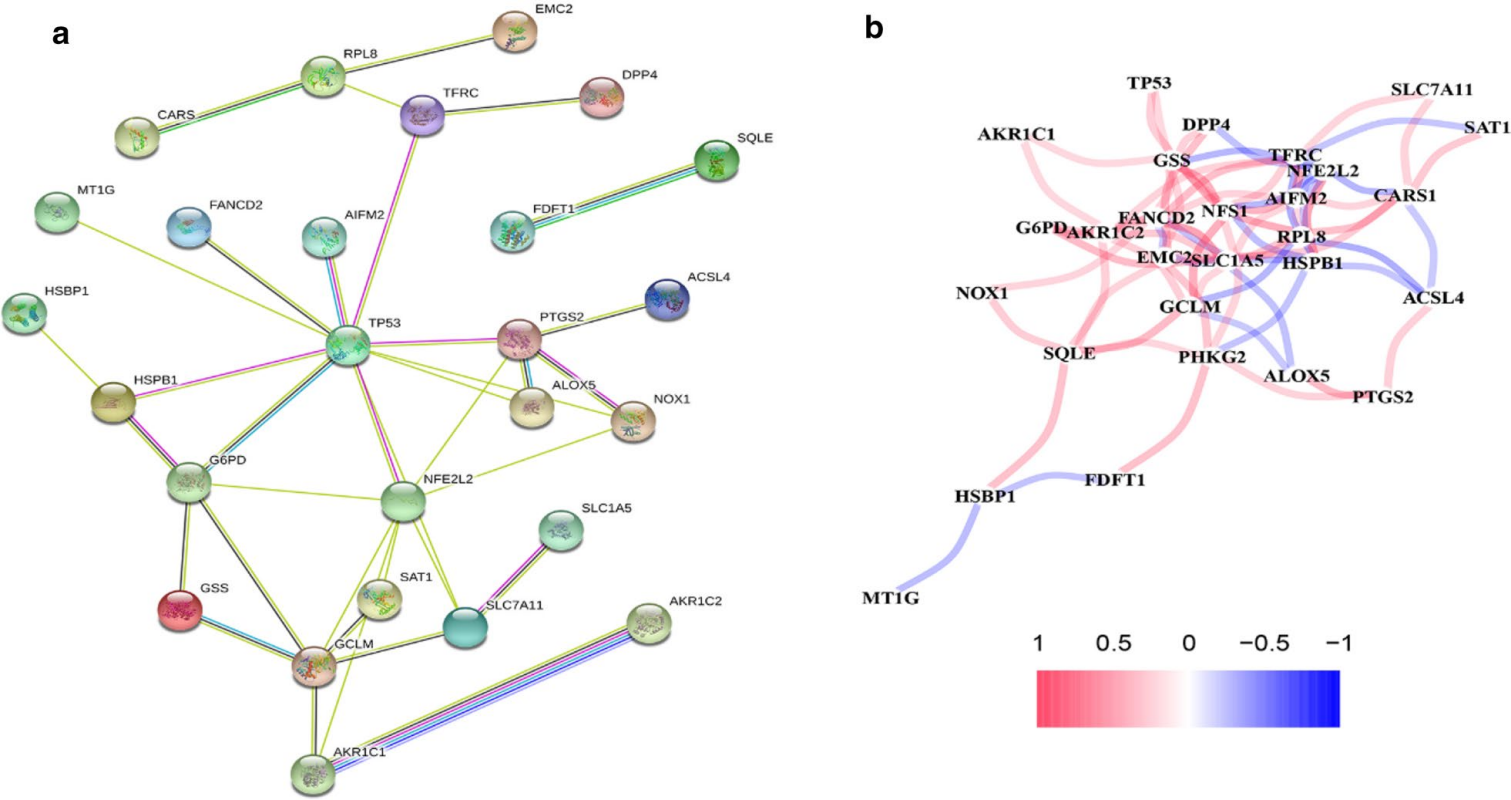
Gene interactions and correlations plots of SDG. **a** The gene network downloaded from the STRING database indicates the interactions among the SDG. **b** The correlation network of genes. Red line represents the positive correlation, while the blue line represents the negative correlation

We explored the SDG interaction at the STRING online website, and the gene network demonstrated the TP53, G6PD, NFE2L2 and PTGS2 were the hub genes (Fig. 3a). The correlations between these SDG are presented in Fig. 3b.

### Prognostic FRG and independent risk factors

**Fig. 4:**
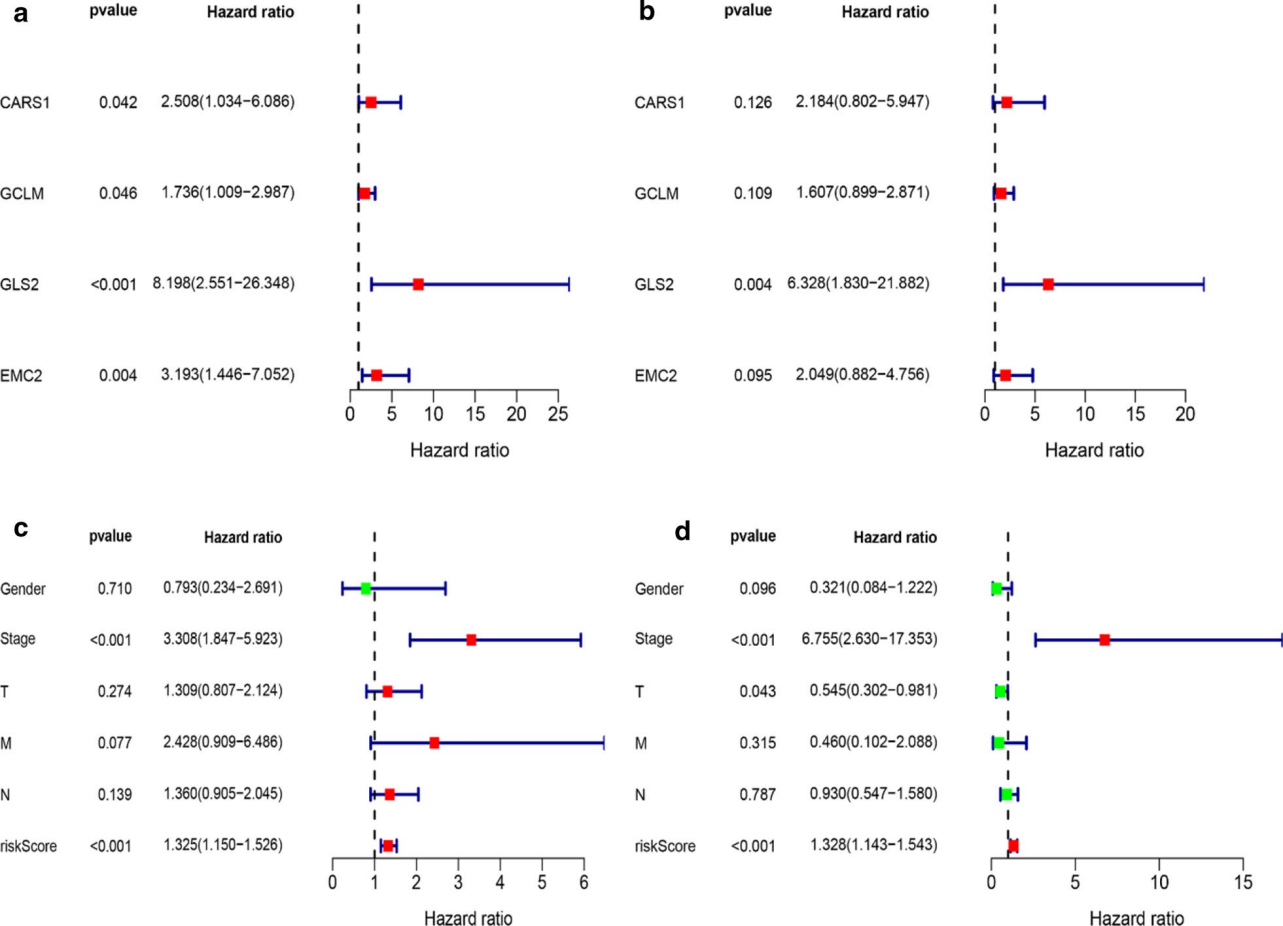
Results of the univariate and multivariate cox analysis of the OS in EAC patients. FRG prognostic values in the univariate (**a**) and multivariate cox analysis (**b**). Risk factors analysis of OS in the univariate (**c**) and multivariate cox regression (**d**). *HR* hazard ratio

Nearly half of the FRG (46.67%, 28/60) were differently expressed in the normal and EAC tissues. By performing the univariate cox regression analysis in the 78 EAC patients, we identified four FRG (CARS1, GCLM, GLS2 and EMC2) were significantly associated with OS (all P < 0.05) (Fig. 4a). Subsequently, multivariate cox regression analysis indicated GLS2 was independent prognostic risk factor (HR = 6.328, P = 0.004) (Fig. 4b).

According to the median of the risk score (risk score formula = 0.781 * expression level of CARS1 + 0.474 * expression level of GCLM + 1.845 * expression level of GLS2 + 0.717 * expression level of EMC2), patients were stratified into high and low-risk groups. Then, we explored the clinical information values in the patients’ OS combining with the FRG. In the univariate cox analysis, we found that tumor stage and risk score were significantly associated with OS (all P < 0.001) (Fig. 4c). And the multivariate cox regression showed the tumor stage and risk score were independent risk factors in EAC patients’ OS (HR = 6.755, HR = 1.328, all P < 0.001 respectively) (Fig. 4d).

### Prognostic hazard curves in high and low-risk patients

**Fig. 5:**
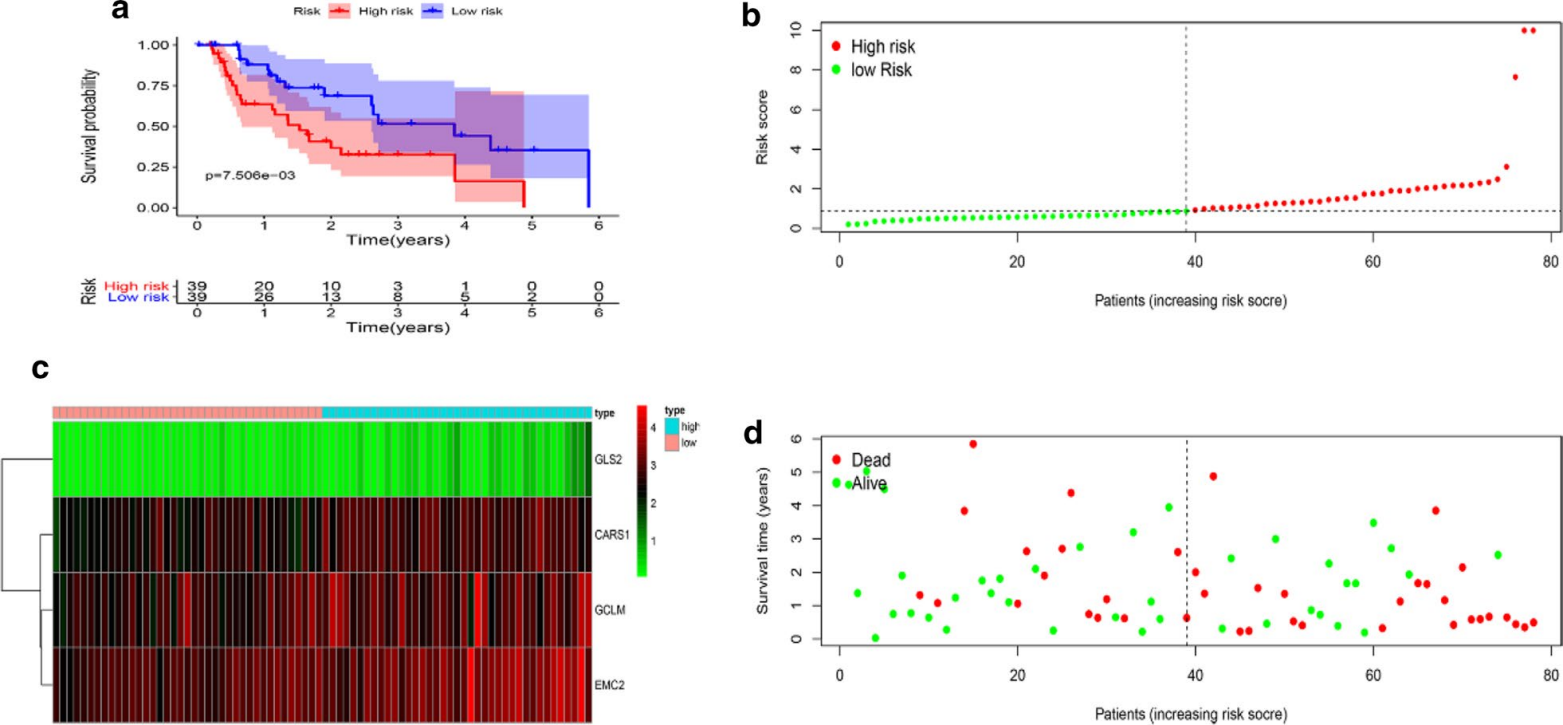
Kaplan–Meier curve and prognostic hazard curves. Kaplan–Meier survival curve (**a**). Risk score curve plot (**b**). The dotted line indicates the individual inflection point of the risk score curve, by which the patients are categorized into low-risk (green) and high-risk (red) groups. Risk score heatmap (**c**). The colors from green to red indicate the expression level from low to high. Risk score scatter plot of high-risk and low-risk (**d**). Red dots indicate the dead patients and green dots indicate the alive. With the increase of risk score, more patients died

Seventy-eight EAC patients were divided into high-risk group (n = 39) and low-risk patients (n = 39) according to the median of the risk score. The Kaplan–Meier curve shows patients with high-risk score have a significant higher death probability than those with low-risk (median time = 0.657 years vs. 1.192 years, p = 0.0075, Fig. 5a). As the risk score increases, the patients’ death risk increases, AND the survival time decreases (Fig. 5b, d). The risk heatmap clearly shows EMC2 was up-regulated in high-risk group compared with the low-risk group, implying it is a tumor-promoting role (Fig. 5c).

### Construction of predictive models

In order to provide an applicable method to predict the EAC patients’ OS, we established the ROC curve using the independent risk factor (risk score) from the multivariate cox regression.

**Fig. 6:**
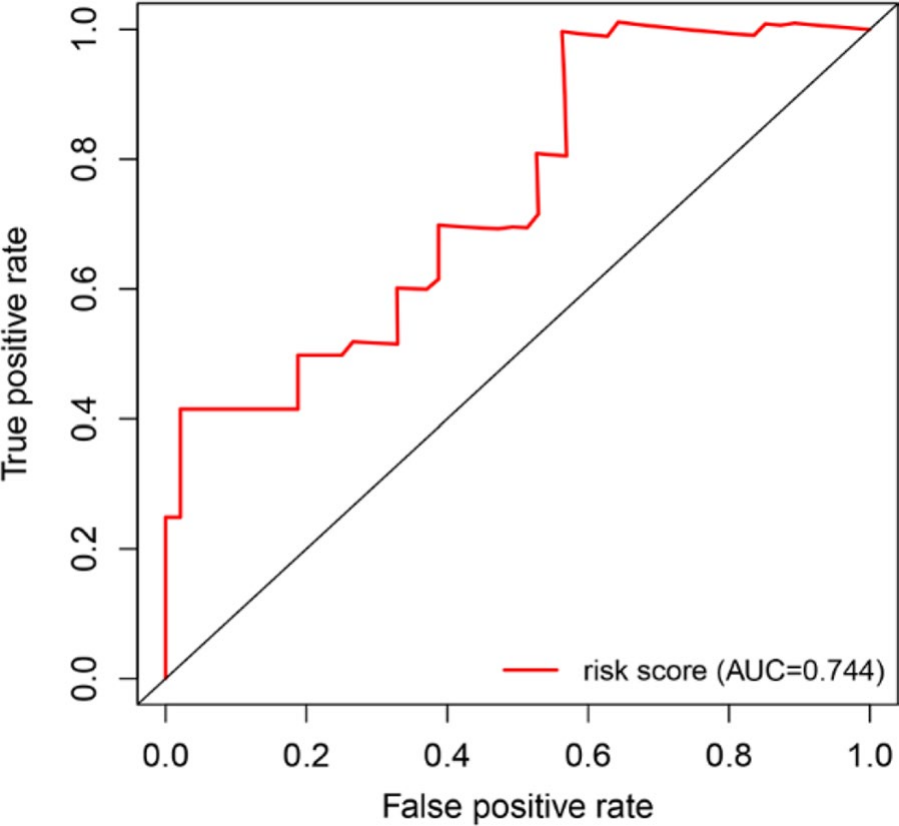
ROC curve of risk score. The AUC ranges from 0.5 to 1.0, with near 1.0 indicating perfect predictive ability. The horizontal axis shows false positive rate, and vertical axis shows true positive rate

In addition, we also assessed the feasibility using the area under curve (AUC) value. The results showed the risk score had better predictive ability (AUC = 0.744) (Fig. 6).

### Immune cell enrichment analysis

**Fig. 7:**
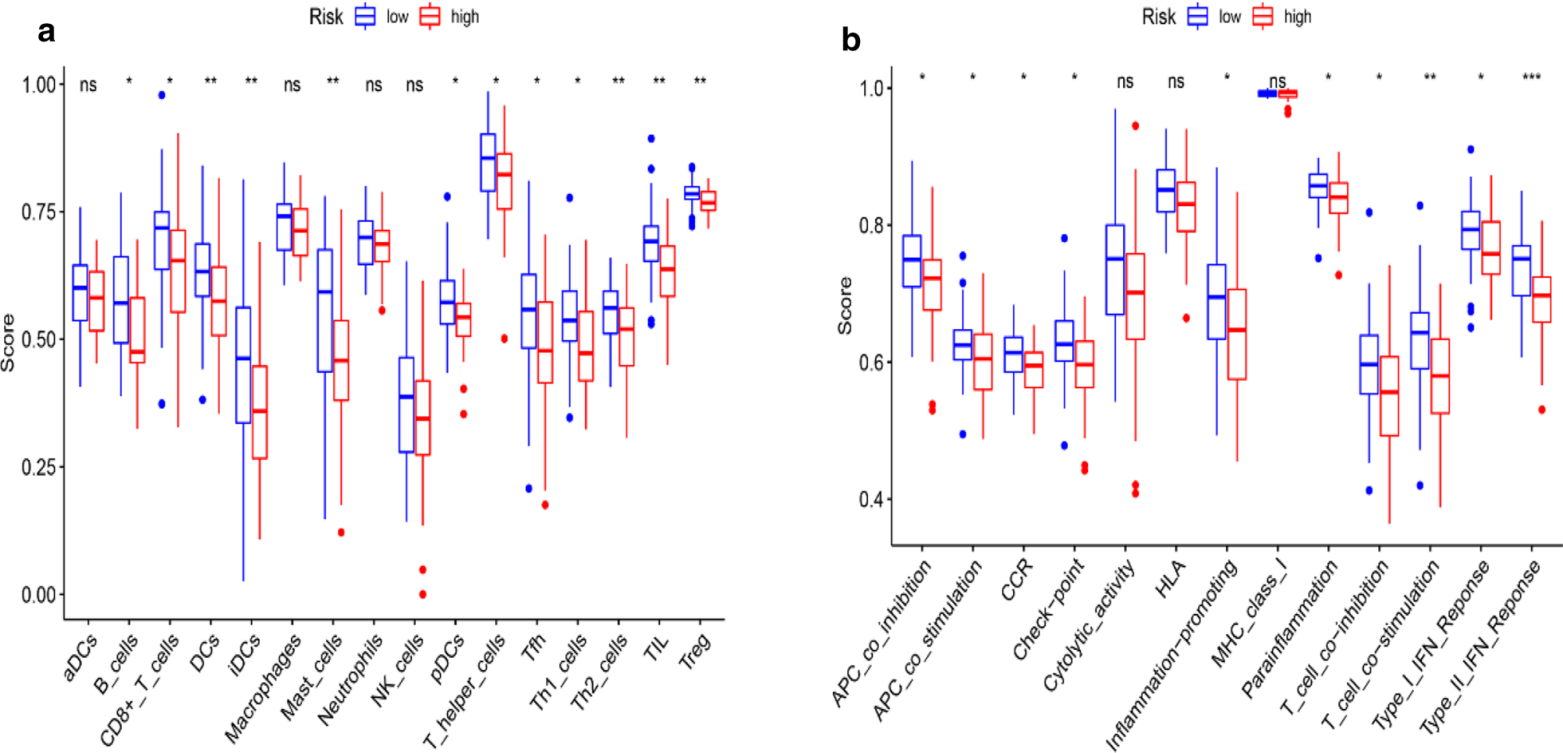
Comparison of the ssGSEA scores between the high-risk and low-risk groups. The scores of 16 immune cells (**a**) and 13 immune-related functions (**b**) are displayed in boxplots. *DCs* dendritic cells, *iDCs* immature DCs, *pDCs* plasmacytoid dendritic cells, *TIL* tumor infiltrating lymphocyte, *CCR* cytokine-cytokine receptor, *APC* antigen presenting cells. Adjusted P values were shown as: ns, not significant; *P < 0.05; **P < 0.01; ***P < 0.001

To further explore the relationships between the risk scores and immune cells and functions, we quantified the enrichment scores of 16 immune cell subpopulations and their related functions with the ssGSEA R package. The results showed the types of immune cells (such as DCs, iDCs, mast cells, Th2 cells, TIL cell, Treg cells, B cells, CD8+ T cells, pDCs, T helper cells, Th1 and Tfh cells) in the high-risk group were significant different with those in the low-risk group (Fig. 7a). Moreover, the scores of the immune functions, such as the type I IFN response, type II IFN response, T cell co-inhibition, APC inhibition and check-point were significantly higher in low-risk group, implying their immunological functions associated with ferroptosis were more active in the low-risk group (Fig. 7b).

**Fig. 8:**
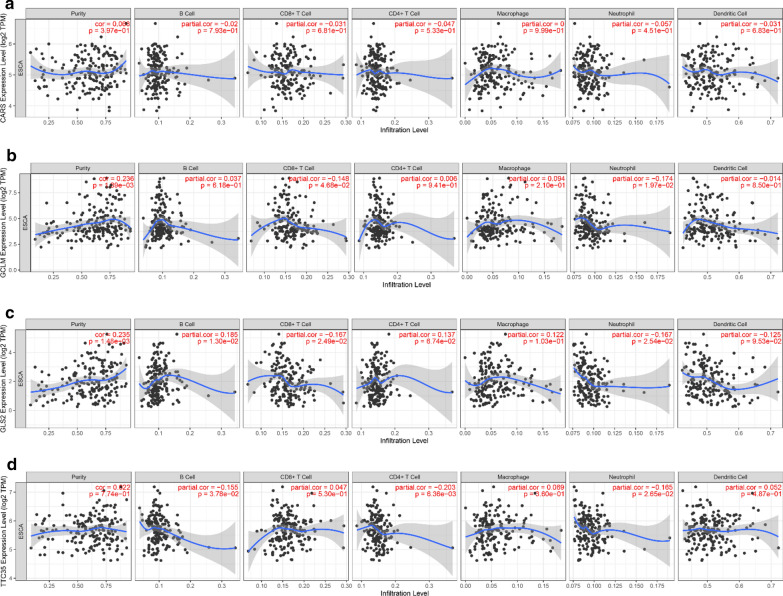
Relations between immune cells and prognostic genes. **a** CARS1 (also referring to CARS) expression level and immune cells in esophageal cancer; **b** GCLM and immune cells; **c** GLS2 and immune cells; **d** EMC2 (alias TTC35) and immune cells. *TPM* transcripts per kilobase million

To better understand characteristics of immune cells and their relations with FRG, the TIMER database was used to analyze the correlation between the abundance of immune cells and the four prognostic genes (CARS1, GCLM, GLS2 and EMC2). The results show GCLM is negatively associated with CD8^+^ T cell and neutrophil (P = 0.047, 0.020 respectively). GLS2 have negative correlations with CD8^+^ T cell and neutrophil (P = 0.025, 0.025 respectively), positive with B cell (P = 0.013). EMC2 (also known as TTC35) is negatively correlated with B cell, CD4^+^ T cell and neutrophil (P = 0.038, 0.006, 0.027 respectively). The details are shown in Fig. 8a–d.

### Clinical experimental validation

**Fig. 9:**
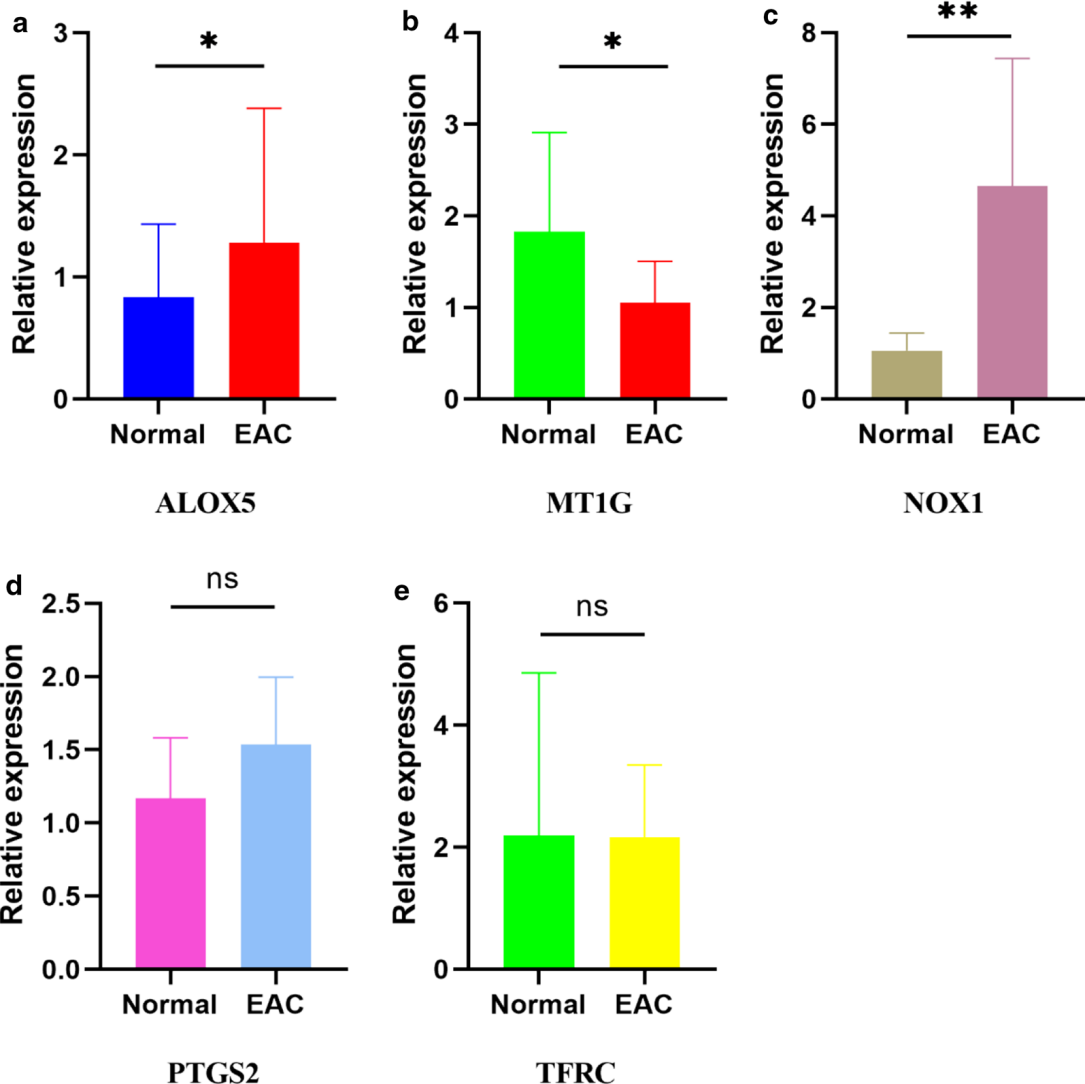
The relative expression levels of the five genes in normal and EAC tissues. The ALOX5 (**a**), NOX1 (**c**) are up-regulated significantly and MT1G (**b**) is down-regulated in the EAC tissues. No significant differences are observed in the PTGS2 (**d**) and TFRC (**e**). *P < 0.05; **P < 0.01, *ns* not significant

We performed the PCR and IHC validation in clinical specimens following the steps described above. We verified the five most significantly different genes according the logFC values (NOX1, MT1G, PTGS2, ALOX5, TFRC). By analysis, the PCR results showed MT1G was down-regulated and the ALOX5 and NOX1 were significantly up-regulated in the EAC tissues. There were no significant differences in the expression of PTGS2 and TFRC between the normal and EAC specimens. The details of the five genes were visualized in Fig. 9a–e (Additional file 6).

**Fig. 10:**
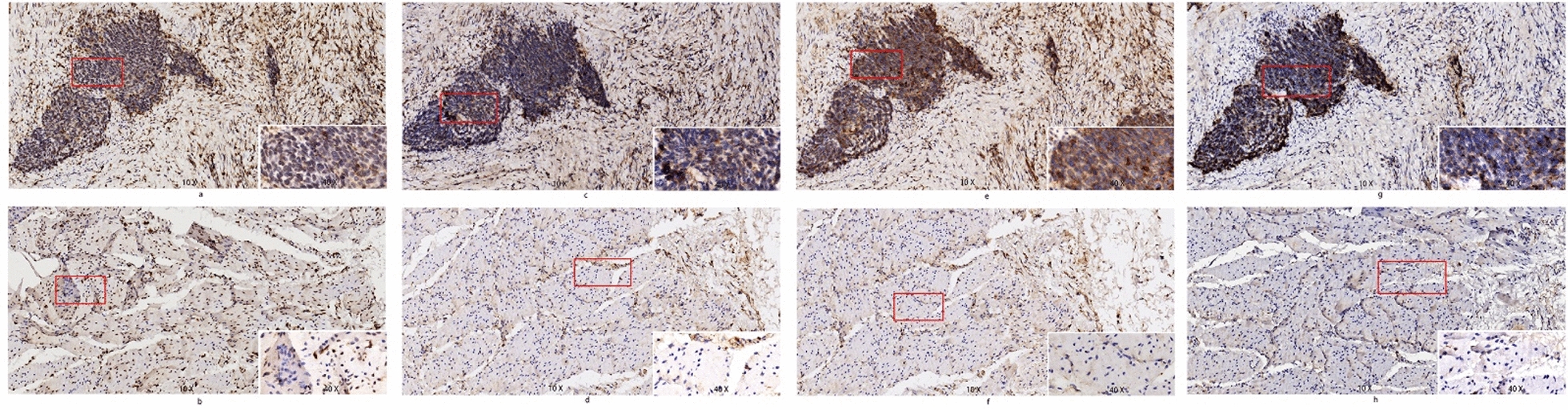
Representative immunohistochemistry results, patient 16#. The upper row (**a**, **c**, **e**, **g**) represents tumor tissue, the lower row (**b**, **d**, **f**, **h**) represents normal tissue. **a**, **b** Protein of ALOX5; **c**, **d** Protein of NOX1; **e**, **f** Protein of PTGS2; **g**, **h** protein of TFRC. The IHC results demonstrated the proteins of ALOX5, NOX1, PTGS2 and TFRC were evidently at high expression level in EAC tissues. ×10: scale bar = 200 µm; ×40: scale bar = 50 µm

Immunohistochemistry results showed ALOX5, NOX1, PTGS2 and TFRC proteins were expressed at high frequency in EAC tissues compared to normal tissues (Fig. 10a–h). Interestingly, the PTGS2 and TFRC protein levels were not consistent with the PCR results. This may be related to the gene transcription regulation and small sample size. The complete IHC results are provided in Additional file 7: Figure S1.

## Discussion

Cell death is a vital necessity for maintaining homeostasis, development and the prevention of excessively proliferative malignancy, such as cancer. To sustain the infinite self-renewal capacity, cancer cells exhibit the overwhelming demands for supply, including the energy metabolism, (anti)oxidant modification and iron intake [14]. The iron-dependent mechanism makes cancer cells more susceptible to iron-catalyzed necrosis, namely ferroptosis [7]. The proposal of ferroptosis has challenged the previous dogma that almost cell deaths were subject to the caspase-dependent apoptosis. Ferroptosis highlights the significance of iron metabolism and creates a new promising area in cancer management. This unique type of death has attracted considerable studies exploring the ferroptotic potential mechanisms and pathways in various cancers [19–21]. However, the specific role of ferroptosis in EAC is unclear. In the current study, we systematically investigated the ferroptosis-related genes expression profiles in EAC. We found that almost half of genes (46.67%, 28/60) were expressed obviously different between the EAC and normal tissues. Functional enrichment analyses results showed these genes were mainly associated with iron-related pathways, such as metabolic and oxidative process. Survival analysis showed four genes (CARS1, GCLM, GLS2 and EMC2) had prognostic values. In addition, the immune cell enrichment analysis revealed the ferroptosis had a close connection with tumor immunity. These findings strongly implied the great potential roles of ferroptosis in EAC.

The essence of ferroptosis is a metabolic necrosis triggered by an iron-catalyzed excessive peroxidation of polyunsaturated fatty acids (PUFAs) [7]. Non-enzymatic lipid peroxidation or auto-oxidation of lipids is indispensable for the initiation of the PUFAs oxidation [22]. Beyond these, enzymatic lipid peroxidation is another chain reaction to catalyze the PUFAs mediated by lipoxygenase (LOX) family [15, 23]. The toxic consequence of continuous oxidation is the loss of the membrane integrity, leading to the occurrence of ferroptosis ultimately. Consistent with these facts, functional enrichment analysis of this study demonstrated these different expressed FRG were mainly associated with the oxidative and iron-related reactions. In addition, the GO and KEGG results also showed there was a close link with GSH metabolism and biosynthesis. GSH, an anti-oxidant, could regulate the sensitivity and resistance of ferroptosis by serving as a cofactor for GPX4 (a member of enzymes) to reduce the lipid hydroperoxides [24, 25]. GSH could be able to reduce the accumulation of phospholipid hydroperoxides and responsible for the detoxification. Direct or indirect inhibition of GSH can induce the initiation of ferroptosis [8]. Therefore, it’s conceivable that, anti-cancer therapy that targeting GPX4 and/or GSH may bring satisfactory effect.

The survival analysis and prognostic ROC models were developed based on four genes (CARS1, GCLM, GLS2 and EMC2) in this study. The FRG can be roughly classified into four categories according to their functions in ferroptosis: iron metabolism, lipid metabolism, (anti)oxidant metabolism (CARS1, GCLM) and energy metabolism (GLS2, EMC2) [14, 15]. Among these, GLS2 (Glutaminase) is an independent risk factor for OS in patients with EAC in our study. The human GLS2 gene is located in chromosome 12, consisting of 18 kb and 18 exons [26]. The GLS2 could regulate the biosynthesis of GSH during the process of ferroptosis and serve as a target of p53 gene [27]. It’s been confirmed that GLS2 has complex connections with cancers, and the up- or down-regulated expression level is significantly associated with patients’ survival in different cancers [28–30]. Hence, it’s easy to understand that the roles of GLS2 are likely to be tumor type-specific, and it enhances the notion that the implication of GLS2 in ferroptosis needs to be carefully interpreted in a context-dependent manner. These studies are in line with our results, pointing out GLS2 has positive correlation with patients’ prognosis. It’s reasonable to hypothesize the abnormal of GLS2 could promote the development of tumor through regulating the ferroptotic pathway. Although the mechanisms by which the GLS2 mediates the ferroptosis and leads to cancer remain elusive, Niu et al. [31] gave us a hint that the occurrence of cancer may be a consequence of ferroptosis regulated by the miRNA/GLS2 axis.

GSH is the most abundant antioxidant in the cell, which synthetizes from glutamate, cysteine and glycine [14, 32]. GCLM, also known as glutamate-cysteine ligase modifier, is the first-rate limiting enzyme of GSH synthesis. The inhibition of GCLM will induce the ferroptosis. The gene expression profiles show the GCLM level is up-regulated in many tumors [33, 34]. Moreover, the GCLM expression level is negatively associated with patients’ relapse-free survival (RFS) and OS [33]. Our analysis is consistent with these findings, suggesting the GCLM is an oncogene (HR > 1) in EAC. The existence of GLCM that drives ferroptosis has important implications for cancer therapy. A recent study by Sharma P identified the Andrographis, a medicine herb, could overcome the colorectal cancer chemoresistance by regulating the ferroptosis genes, such as GCLM [34]. Therefore, drugs that target ferroptosis can be exploited and provide an efficient strategy for clinical application. CARS1 alias: CARS (cysteinyl-tRNA synthetase), is still at infancy in the area of ferroptosis. Study shows that knockdown of CARS1 could cause increased transsulfuration pathway activity, and resistance to ferroptosis [14, 15]. EMC2, also referring to TTC35, is ER (endoplasmic reticulum) membrane protein complex subunit 2. This gene shares similarities with other ferroptosis-related genes, and its knockdown suppresses erastin-induced ferroptosis [14].

The notion that immunity promotes or suppresses the tumor is well accepted, and one of the most impactful anti-cancer therapies developed in recent years is the immune checkpoint therapy. Our results demonstrated that the immune statue was significantly different between the low-risk and high-risk EAC patients, including the DCs, CD8+ T cells, type I IFN response, type II IFN response et.al. In addition, the prognostic genes (GCLM, GLS2) have significant correlations with CD8+ T cells, indicating the complexity between ferroptosis and immunity. The enigmatic and sophisticated relations linking immunity with ferroptosis are being gradually revealed with the progress of the experimental trials in vivo and vitro. Researchers found DCs in tumor-bearing hosts accumulate plenty of lipids and PUFAs, causing the impaired ability to present the antigen and stimulate the inadequate CD8+ T cells responses [35, 36]. This lends support to the idea that DCs and CD8+ T cells contribute to the ferroptosis through regulating the lipids and PUFAs. As expected, experiment ex vivo demonstrated that T cell lipid peroxidation could induce ferroptosis and prevent immunity to infection in the study by Matsushita et al. [37]. Consistent with the results of Matsushita et al. preclinical models have confirmed CD8+ T cells could enhance ferroptosis-specific lipid peroxidation and increase ferroptosis by releasing the IFN-γ (II IFN), thus increasing the efficacy of immunotherapy [12]. In this study, we found the contents of DCs and CD8+ T cells were significantly higher in the low-risk group than those in high-risk group. One plausible explanation is DCs and CD8+ T cells activate the ferroptosis process by releasing signals, such as IFN-γ. Additionally, we also found the type I IFN (IFN-α, β) was also higher in the low-risk group, indicating the type I IFN may be necessary to initiate the ferroptosis. However, it’s undeniable that more work is warranted to confirm the above results.

The strength of this study is that we performed a systematically analysis based on the national database for the first time, and summarized the current knowledge about the ferroptosis genes in the EAC. Notably, it should be aware that the methods provided in this study did not meet all the requirements for the gene expression levels. Meanwhile, there are some limitations in our study. Firstly, the clinical information downloaded from the TCGA is incomplete, especially the therapy, which may be helpful to understand whether FRG are biomarkers of treatment. Secondly, the mechanism how ferroptosis modulates the precise process of EAC is unclear. Lastly, the prognostic model needs to be verified in a large-scale and multicenter clinical cohort. Notwithstanding its limitations, this study does provide a comprehensive overview of FRG profiles in EAC and these limitations can be solved if there are enough data in the future.

## Conclusions

In conclusion, we identified differently expressed ferroptosis-related genes that may involve in the process in EAC. These genes have significant values in predicting the patients’ OS and utilizing ferroptosis may be the therapeutic targets. Further studies are necessary to verify these results in our study.

## Supplementary Information

**Additional file 1: Table S1.** Ferroptosis-related genes and their full names

**Additional file 2: Table S2.** The annotation of 16 immune cells and 13 functions in ssGSEA.

**Additional file 3: Table S3.** Primers design and their sequences of ferroptosis.

**Additional file 4.** The gene expression data of EAC patients from TCGA

**Additional file 5.** Clinical information of EAC patients

**Additional file 6.** Raw data of PCR results in EAC patients

**Additional file 7: Figure S1.** Immunohistochemistry results.

## Data Availability

All data were available in TCGA database (https://portal.gdc.cancer.gov). All the experimental data analyzed and displayed in the present manuscript are available from the corresponding author upon reasonable request.

## Abbreviations

EAC: Esophageal adenocarcinoma
ROS: Reactive oxygen species
GSH: Glutathione
FRG: Ferroptosis-related genes
PCR: Polymerase chain reaction
IHC: Immunohistochemistry
TCGA: The Cancer Genome Atlas
SDG: Significantly different genes
FDR: False discovery rate
OS: Overall survival
GO: Gene Ontology
KEGG: Kyoto Encyclopedia of Genes and Genomes
ROC: Receiver Operating Characteristic
AUC: Area under curve
ssGSEA: Single-sample gene set enrichment analysis
cDNA: Complementary DNA
gDNA: Genomic DNA
GAPDH: Glyceraldehyde-3-phosphate dehydrogenase
SA-HRP: Streptavidin–horseradish peroxidase
DAB: 3,3′-Diaminobenzidine tetrahydrochloride
HR: Hazard ratio
DCs: Dendritic cells
iDCs: Immature DCs
pDCs: Plasmacytoid dendritic cells
TIL: Tumor infiltrating lymphocyte
CCR: Cytokine–cytokine receptor
APC: Antigen presenting cells
TPM: Transcripts per kilobase million
PUFAs: Polyunsaturated fatty acids
LOX: Lipoxygenase
GLS2: Glutaminase
RFS: Relapse-free survival
