# Supplementary material for: Identification the ferroptosis-related gene signature in patients with esophageal adenocarcinoma
Source: Cancer Cell Int. 2021 Feb 18;21:124. doi: 10.1186/s12935-021-01821-2 (PMC7891153; doi:10.1186/s12935-021-01821-2)
Supplement: Supplementary file 3 — Additional file 3: Table S3. Primers design and their sequences of ferroptosis. [file 12935_2021_1821_MOESM3_ESM.docx]

**Table S3 Primers design and their sequences of ferroptosis**

| **Number** | **Gene Primer** | **Base sequence (5'to 3')** | **Base number** | **Purification method** |
| --- | --- | --- | --- | --- |
| 1 | ACSL4-F1 | GTCCGCATGATGCTGTCT | 18 | tPAGE |
| 2 | ACSL4-R1 | CTCCTGCTTGTAACTTCACT | 20 | tPAGE |
| 3 | AIFM2-F1 | GTGAGCAATCTGGAGGAGC | 19 | tPAGE |
| 4 | AIFM2-R1 | GGACAGGAGGAACGTCAGT | 19 | tPAGE |
| 5 | AKR1C1-F1 | CCACCGAGAAGAACCATG | 18 | tPAGE |
| 6 | AKR1C1-R1 | TGAAATCACCAAGCAGGA | 18 | tPAGE |
| 7 | AKR1C2-F1 | CTGGGATTTGGCACCTAT | 18 | tPAGE |
| 8 | AKR1C2-R1 | GACATGCAATCACGGAAG | 18 | tPAGE |
| 9 | ALOX5-F1 | CCAAGTCGGTCAAGAGCC | 18 | tPAGE |
| 10 | ALOX5-R1 | TACATGCCCAGGAACAGC | 18 | tPAGE |
| 11 | CARS1-F1 | TTGAAGACCACGAAGGAC | 18 | tPAGE |
| 12 | CARS1-R1 | CCAAGGGTGACTGTAAACAT | 20 | tPAGE |
| 13 | DPP4-F1 | AGTGGCGTGTTCAAGTGT | 18 | tPAGE |
| 14 | DPP4-R1 | AGTGGCTCATGTGGGTAT | 18 | tPAGE |
| 15 | EMC2-F1 | TAGACTATGGTCGGGATG | 18 | tPAGE |
| 16 | EMC2-R1 | CAAGTCCACCTTGGGTAT | 18 | tPAGE |
| 17 | FANCD2-F1 | ACACGAGACTCACCCAACA | 19 | tPAGE |
| 18 | FANCD2-R1 | AGCGGATTTCAAACTCTAACAC | 22 | tPAGE |
| 19 | FDFT1-F1 | GAGGTTTGGAGCAGGTAT | 18 | tPAGE |
| 20 | FDFT1-R1 | GTGGCTTCGGGAAATCAG | 18 | tPAGE |
| 21 | G6PD-F1 | AGCCGTCGTCCTCTATGTGG | 20 | tPAGE |
| 22 | G6PD-R1 | CGTTCTTGTATCTGTTGCCGTA | 22 | tPAGE |
| 23 | GCLM-F1 | ACAGCGAGGAGGAGTTTC | 18 | tPAGE |
| 24 | GCLM-R1 | CAGCAGGAGGCAAGATTA | 18 | tPAGE |
| 25 | GSS-F1 | ACTCTTTCCAGCACCATCA | 20 | tPAGE |
| 26 | GSS-R1 | GAGCAATCAGTAGCACCAGA | 20 | tPAGE |
| 27 | HSBP1-F1 | GAAGTGTAGGTTACGGTCTG | 20 | tPAGE |
| 28 | HSBP1-R1 | AAGGTCCAAGATGTGAAGA | 19 | tPAGE |
| 29 | HSPB1-F1 | CAAGGATGGCGTGGTGGA | 20 | tPAGE |
| 30 | HSPB1-R1 | CGAAGGTGACTGGGATGGTGA | 20 | tPAGE |
| 31 | MT1G-F1 | TCTTCCCTTCTCGCTTGG | 18 | tPAGE |
| 32 | MT1G-R1 | TTACGGGTCACTCTATTTGTAC | 22 | tPAGE |
| 33 | NFE2L2-F1 | ATTCCTTCAGCAGCATCC | 18 | tPAGE |
| 34 | NFE2L2-R1 | CTTCCAGGGGCACTATCT | 18 | tPAGE |
| 35 | NFS1-F1 | AGTATGACCACAAGCGAATC | 20 | tPAGE |
| 36 | NFS1-R1 | ATGCCATCCTGAACCATC | 18 | tPAGE |
| 37 | NOX1-F1 | GAGATGGAGGAATTAGGC | 18 | tPAGE |
| 38 | NOX1-R1 | AATACCAGGGAGTCAAGG | 18 | tPAGE |
| 39 | PHKG2-F1 | TACCAGAAGTACGACCCTAA | 20 | tPAGE |
| **Number** | **Gene Primer** | **Base sequence (5'to 3')** | **Base number** | **Purification method** |
| 40 | PHKG2-R1 | GAACCCGAAATCTGAAAGT | 19 | tPAGE |
| 41 | PTGS2-F1 | TCAGCCATACAGCAAATC | 18 | tPAGE |
| 42 | PTGS2-R1 | CATCAGGCACAGGAGGAA | 18 | tPAGE |
| 43 | RPL8-F1 | CTACATCAAGGGCATCGTCA | 20 | tPAGE |
| 44 | RPL8-R1 | TCCGTCCGCTTCTTAAACC | 19 | tPAGE |
| 45 | SAT1-F1 | GAACCACCTCCTCCTACT | 18 | tPAGE |
| 46 | SAT1-R1 | CTATGCCAAAGCCTCTAT | 18 | tPAGE |
| 47 | SLC1A5-F1 | GCCTTTCGCTCATACTCTACC | 21 | tPAGE |
| 48 | SLC1A5-R1 | CCACATCCTCCATCTCCAC | 19 | tPAGE |
| 49 | SLC7A11-F1 | GGTCAGAAAGCCTGTTGT | 18 | tPAGE |
| 50 | SLC7A11-R1 | GTTCCACCCAGACTCGTA | 18 | tPAGE |
| 51 | SQLE-F1 | AGAAGTTATCATCGTGGGA | 19 | tPAGE |
| 52 | SQLE-R1 | ACAACAGTCAGTGGAGCA | 18 | tPAGE |
| 53 | TFRC-F1 | TCAGTTTCCACCATCTCG | 18 | tPAGE |
| 54 | TFRC-R1 | AAGTCTCCAGCACTCCAA | 18 | tPAGE |
| 55 | TP53-F1 | ACCCAGGTCCAGATGAAG | 18 | tPAGE |
| 56 | TP53-R1 | CACTCGGATAAGATGCTGA | 19 | tPAGE |
