# Supplementary material for: Identification the ferroptosis-related gene signature in patients with esophageal adenocarcinoma
Source: Cancer Cell Int. 2021 Feb 18;21:124. doi: 10.1186/s12935-021-01821-2 (PMC7891153; doi:10.1186/s12935-021-01821-2)
Supplement: Supplementary file 7 — Additional file 7: Figure S1. Immunohistochemistry results. [file 12935_2021_1821_MOESM7_ESM.docx]

**Figure S1. Immunohistochemistry results**


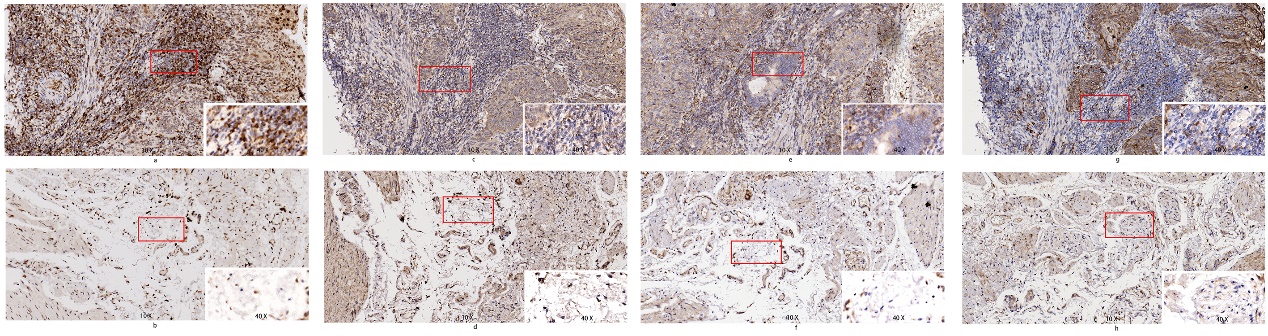


Immunohistochemistry results, patient 1#. The upper row (a, c, e, g) represents tumor tissue, the lower row (b, d, f, h) represents normal tissue. (a, b) Protein of ALOX5; (c, d) Protein of NOX1; (e, f) Protein of PTGS2; (g, h) Protein of TFRC. The IHC results demonstrated the proteins of ALOX5, NOX1, PTGS2 and TFRC were evidently at high expression level in EAC tissues. 10 X: scale bar=200 um; 40 X: scale bar=50 um.


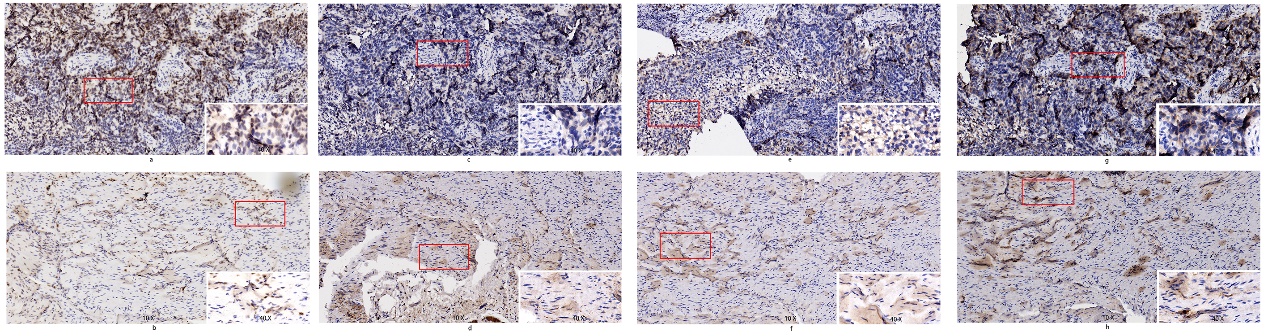


Immunohistochemistry results, patient 6#. The upper row (a, c, e, g) represents tumor tissue, the lower row (b, d, f, h) represents normal tissue. (a, b) Protein of ALOX5; (c, d) Protein of NOX1; (e, f) Protein of PTGS2; (g, h) Protein of TFRC. The IHC results demonstrated the proteins of ALOX5, NOX1, PTGS2 and TFRC were evidently at high expression level in EAC tissues. 10 X: scale bar=200 um; 40 X: scale bar=50 um.


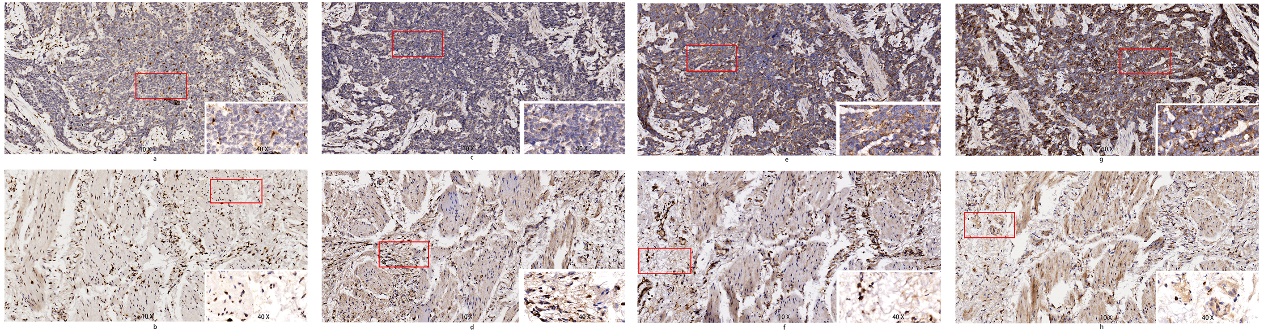


Immunohistochemistry results, patient 9#. The upper row (a, c, e, g) represents tumor tissue, the lower row (b, d, f, h) represents normal tissue. (a, b) Protein of ALOX5; (c, d) Protein of NOX1; (e, f) Protein of PTGS2; (g, h) Protein of TFRC. The IHC results demonstrated the proteins of ALOX5, NOX1, PTGS2 and TFRC were evidently at high expression level in EAC tissues. 10 X: scale bar=200 um; 40 X: scale bar=50 um.


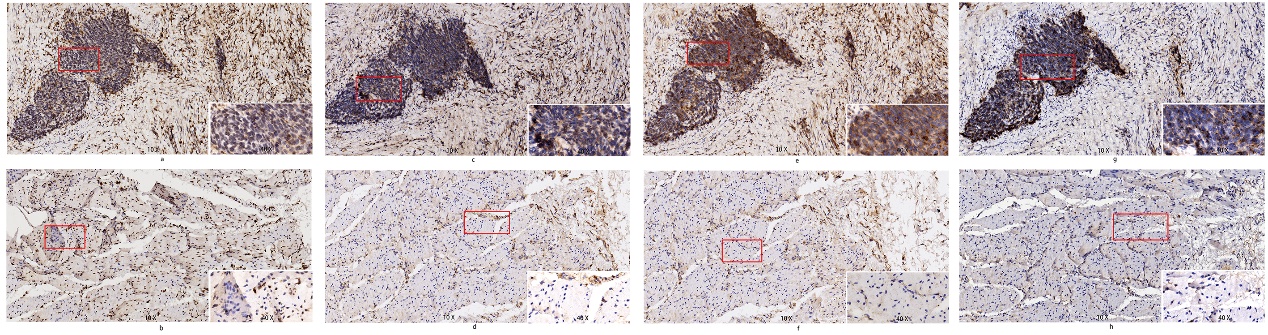


Immunohistochemistry results, patient 16#. The upper row (a, c, e, g) represents tumor tissue, the lower row (b, d, f, h) represents normal tissue. (a, b) Protein of ALOX5; (c, d) Protein of NOX1; (e, f) Protein of PTGS2; (g, h) Protein of TFRC. The IHC results demonstrated the proteins of ALOX5, NOX1, PTGS2 and TFRC were evidently at high expression level in EAC tissues. 10 X: scale bar=200 um; 40 X: scale bar=50 um.


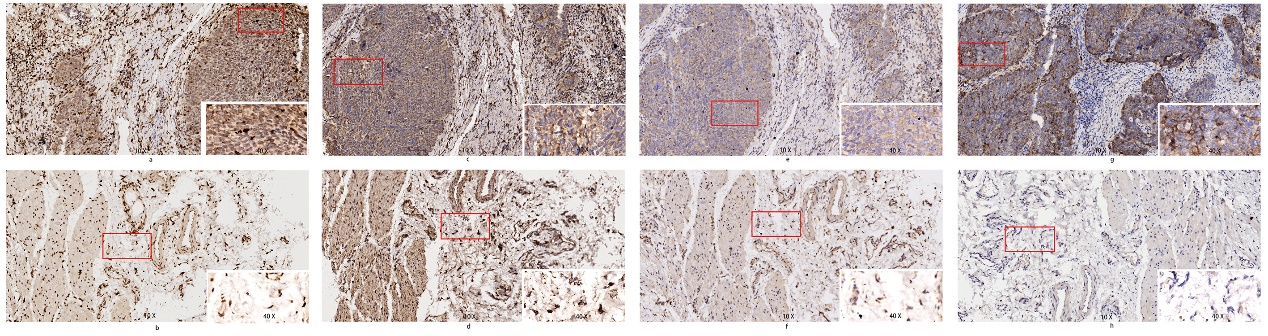


Immunohistochemistry results, patient 19#. The upper row (a, c, e, g) represents tumor tissue, the lower row (b, d, f, h) represents normal tissue. (a, b) Protein of ALOX5; (c, d) Protein of NOX1; (e, f) Protein of PTGS2; (g, h) Protein of TFRC. The IHC results demonstrated the proteins of ALOX5, NOX1, PTGS2 and TFRC were evidently at high expression level in EAC tissues. 10 X: scale bar=200 um; 40 X: scale bar=50 um.
